# Theoretical Investigation of Photon Interaction and X-Ray Imaging Performance of PEEK-Based Composites for Medical Implants

**DOI:** 10.3390/polym17070996

**Published:** 2025-04-07

**Authors:** Hanan Akhdar

**Affiliations:** Department of Physics, Faculty of Science, Imam Mohammad Ibn Saud Islamic University (IMSIU), P.O. Box 90950, Riyadh 11623, Saudi Arabia; hfakdar@imamu.edu.sa

**Keywords:** PEEK, medical implants, photon attenuation, X-ray imaging quality, EpiXS, Monte Carlo simulation, Geant4

## Abstract

Polyetheretherketone (PEEK) is a high-performance, biocompatible polymer with remarkable mechanical properties, making it a promising candidate for medical implants. However, its intrinsic radiolucency poses a challenge for post-operative imaging. This study investigates the photon shielding capabilities and X-ray imaging qualities of pure PEEK and its composites with barium sulfate (BaSO_4_), tantalum (Ta), bismuth oxide (Bi_2_O_3_), and hydroxyapatite (HA). The Monte Carlo-based Geant4 toolkit and the EpiXS application were used to evaluate key photon interaction parameters, including mass attenuation coefficients, effective atomic number (Z_eff_), and effective electron density (N_eff_), as well as the imaging performance metrics such as energy deposition and signal-to-noise ratio (SNR). Results indicate that high atomic number composites significantly enhance PEEK’s photon attenuation and imaging contrast. PEEK-Bi_2_O_3_ exhibited the highest attenuation coefficients and energy deposition, making it the most effective X-ray shielding material. PEEK-Ta provided a balanced performance with enhanced shielding and lower secondary radiation effects, making it suitable for applications requiring both radiopacity and imaging stability. PEEK-BaSO_4_ moderately improved attenuation while maintaining a lower density, offering a trade-off between radiopacity and mechanical properties. Conversely, PEEK-HA demonstrated minimal enhancement in photon attenuation, limiting its effectiveness for radiographic applications. The findings suggest that incorporating high atomic number elements into PEEK significantly enhances its suitability for radiopaque medical implants, allowing for improved post-operative monitoring.

## 1. Introduction

Polyetheretherketone (PEEK) is a semi-crystalline polymer composed of repeating units of oxygen, p-phenylene, and carbonyl groups. It is a high-performance, biocompatible thermoplastic polymer with remarkable mechanical strength and chemical stability, making it favorable for biomedical applications [1]. PEEK, with a density of 1.32 g/cm^3^, possesses unique properties such as an elastic modulus of around 4.0 GPa, which is close to that of cortical bone, excellent tensile and bending strengths, and low wear rates and is lightweight, non-toxic, and has a color similar to natural dentin, which makes it suitable for medical implants and bone replacement devices [1,2,3].

Despite these advantages, PEEK is inherently bioinert, meaning it does not naturally bond with human bone. This limitation has led researchers to explore the incorporation of composites or fillers to enhance its surface, mechanical, and biological properties, thereby improving bone integration while preserving its valuable characteristics [4,5,6]. Such composite modifications may ultimately pave the way for new biomedical devices that could replace traditional metallic implants with improved performance [7].

Another significant challenge in using PEEK for medical implants is its intrinsic radiolucency, which hinders routine post-operative assessment using X-ray imaging. Metals typically exhibit high contrast against surrounding tissues in medical images, whereas polymers like PEEK are naturally radiolucent due to their low X-ray attenuation, allowing X-ray photons to pass through with minimal absorption and reducing implant visibility [8,9]. This low attenuation is a result of the material’s composition of low atomic mass elements (such as carbon, hydrogen, oxygen, and nitrogen) and the corresponding low electron density [10]. Consequently, researchers have also investigated the effects of various composites on the X-ray photon attenuation properties of polymers [11].

Although numerous studies have investigated PEEK composites, most have independently addressed either photon attenuation characteristics or biomechanical performance. Several investigations have confirmed that incorporating high-Z fillers into PEEK improves radiopacity, yet often without assessing the corresponding impact on imaging performance [12]. Several studies have also highlighted enhancements in mechanical properties with bio-fillers [10,11], but the implications for X-ray imaging remain insufficiently explored. These research trends underscore a lack of a unified theoretical framework evaluating both radiation shielding and imaging quality, which motivated the present work. By integrating Monte Carlo simulations with analytical attenuation modeling, this study seeks to provide an assessment of PEEK-based composites for medical implant applications.

By integrating both radiation shielding effectiveness and imaging performance assessments, this study aims to provide a comprehensive understanding of PEEK-based materials with various composites. The findings are expected to contribute to the optimization of implant materials for enhanced medical outcomes, informing future material design strategies that will enable the development of PEEK-based implants offering both mechanical excellence and superior post-operative monitoring capabilities.

## 2. Materials and Methods

### 2.1. Materials

**Figure 1:**
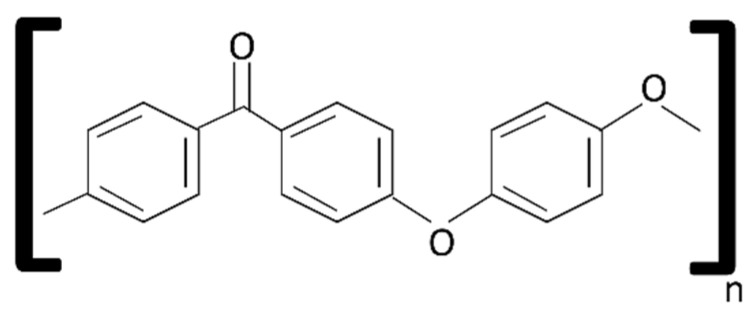
The repeating unit of PEEK.

This study investigates photon shielding abilities and imaging qualities of pure PEEK and PEEK-based composites. Figure 1 illustrates the repeating unit of PEEK.

The studied composites include PEEK with a 20% weight fraction of barium sulfate (BaSO_4_), tantalum (Ta), bismuth oxide (Bi_2_O_3_), and hydroxyapatite (HA). These were selected based on their densities, atomic numbers, and enhancement of radiopacity of materials.

Tantalum (Ta) is a biocompatible metal with a density of 16.65 g/cm^3^, and it is suitable for medical implant applications due to its excellent corrosion resistance and chemical inertness, which can provide reliable and long-lasting medical implants [13]. Barium sulfate (BaSO_4_), with a density of 4.5 g/cm^3^, is increasingly recognized for its value in medical implants because of its excellent radiopacity and chemical inertness [14]. Bismuth oxide (Bi_2_O_3_), with a density of 8.9 g/cm^3^, is known for its uniform particle size distribution and inherent chemical stability, which leads to mechanical strength, thermal resilience, and enhanced radiopacity. These properties make it an advanced biomaterial that combines durability with favorable biocompatibility for future implantable devices [15]. Hydroxyapatite (HA), with a density of 3.16 g/cm^3^, is a biomaterial with intrinsic similarity to the mineral phase of bone, which gives it excellent biocompatibility, osteoconductivity, and bioactivity [16].

Table 1 summarizes the properties of all studied samples, where the values of density and elastic modulus were obtained based on extrapolations from known trends in composite materials. The rule of mixtures was used, as shown in Equation (1) [17].

(1) $$\rho_{Mixture}=f_{composite}\rho_{composite}+f_{PEEK}\rho_{PEEK}$$

### 2.2. Methods

#### 2.2.1. Photon Shielding Properties

EpiXS was used to assess the photon shielding abilities of the investigated samples within the X-ray diagnostic imaging energy range (20–120 keV). EpiXS is a Windows-based application with a very user-friendly interface developed by the Philippine Nuclear Research Institute (PNRI), which allows calculations of photon attenuation parameters by employing the EPICS2017 (ENDF/B-VIII) and EPDL97 (ENDF/B-VI.8) photoatomic libraries to generate mass attenuation coefficients and other essential photon interaction data [18].

The mass attenuation coefficients ($\mu_{m}$) of all studied samples were evaluated. The mass attenuation coefficient of a material is its ability to absorb X-rays and is given by the following equations:

(2) $$\mu_{m}=\frac{\mu }{\rho }$$

(3) $$I=I_{0}e^{-\mu x}$$

where $I_{0}$ is the initial X-ray intensity, I is the transmitted intensity, x is the thickness of the material, and μ is the linear attenuation coefficient.

The half value layer (*HVL*) is also an important parameter, representing the thickness of material required to reduce the X-ray intensity by half, and is given by the following equation:

(4) $$HVL=\frac{ln2}{\mu }$$

Both the effective atomic number (*Z_eff_*) and the effective electron density (*N*_eff_), which quantifies the number of electrons per unit mass available for photon interactions, further influencing X-ray attenuation. The total atomic cross-section can be calculated by Equation (5) [19]:

(5) $$\sigma_{t.a}=\frac{\mu_{m}}{N_{A}\sum_{i}^{n}\left(w_{i}/A_{i}\right)}$$

where (*N_A_*) is Avogadro’s number, and (*A_i_*) is the atomic weight of an element of the compound, while the total electronic cross-section for the element is given by Equation (6) [19]:

(6) $$\sigma_{t.el}=\frac{1}{N_{A}}\sum_{i}^{n}\frac{f_{i}A_{i}}{Z_{i}}{\left(\mu_{mt}\right)}_{i}$$

where (*f_i_*) is the number of atoms of the element (*i*) relative to the total number of atoms of all elements in the compound, (*Z_i_*) is the atomic number of the ith element in the compound. The effective atomic number (*Z_eff_*) of the compound can be found from the ratio between the total atomic cross-section and the total electronic cross-section using Equation (7) [20,21]:

(7) $$Z_{eff}=\frac{\sigma_{t,a}}{\sigma_{t,el}}$$

The effective electron density is given by Equation (8) [20,21]:

(8) $$N_{eff}=\frac{\mu_{m}}{\sigma_{t,el}}$$

#### 2.2.2. X-Ray Imaging Qualities

Imaging quality is quantitatively evaluated through the signal-to-noise ratio (SNR), which can be found using the equation:

(9) $$SNR=\frac{Mean\;Intensity}{Standard\;Deviation}$$

Geant4, a well-known Monte Carlo-based toolkit widely used in nuclear physics, nuclear engineering, and medical physics, was employed to evaluate the X-ray imaging quality of the studied materials [22]. A custom Geant4 code was developed such that the source is defined as a flat plate with a width of 100 cm and a height of 100 cm. The plate source is placed at Z = −50 cm as it is in front of the objects being irradiated. X and Y positions are randomized within the plate area to ensure uniform beam coverage. The X-rays travel along the +Z direction, heading toward the volumes located at Z = 0. The energy range for the X-rays is set between 20 keV and 120 keV, with a random energy selection to simulate X-ray medical imaging energies. This ensures that each event consists of a photon traveling perpendicular to the plate, directly hitting the compared volumes. This means the first face exposed to X-rays is the Z-minimum face (closest to Z = 0), and the back face (Z-positive side) will receive less energy due to absorption and scattering within the material. The four identical volumes are made of bone tissue defined by Geant4 (G4_ICRP BONE) and the investigated PEEK-based composites. The simulations provided detailed insights into energy deposition and enabled the calculation of the signal-to-noise ratio (SNR) from radiographic images. Higher SNR values indicate enhanced image contrast and reduced noise, thereby improving implant visibility. SNR measurements were obtained from images captured in multiple projections (XY, XZ, and YZ) to ensure a comprehensive evaluation of the imaging performance.

Electromagnetic physics models in Geant4 were used to simulate particle interactions across the energy range, including detailed low-energy processes essential for accurate modeling of photon interactions in medical imaging applications. Macrofiles were developed to extract histograms of energy deposition for bone, PEEK, and other investigated materials, then compute the SNR to evaluate the noise levels, and then visualize the results using 2D energy deposition maps.

## 3. Results

### 3.1. Photon Shielding Properties

**Figure 2:**
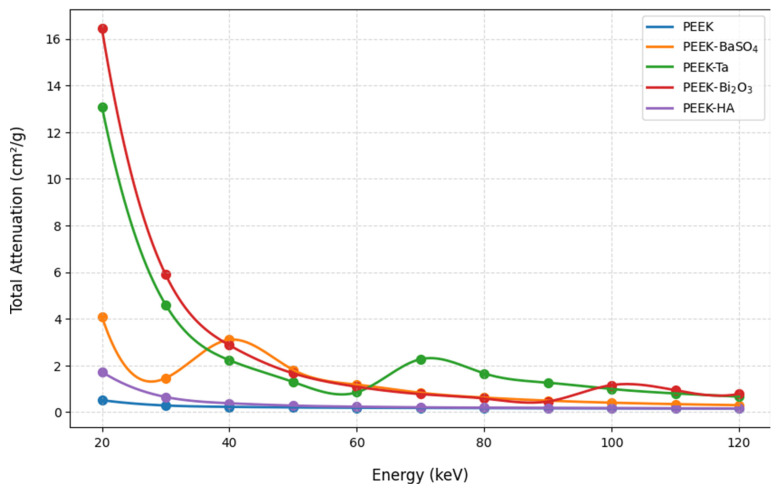
The mass attenuation coefficients of the studied samples within the studied energy range.

**Figure 3:**
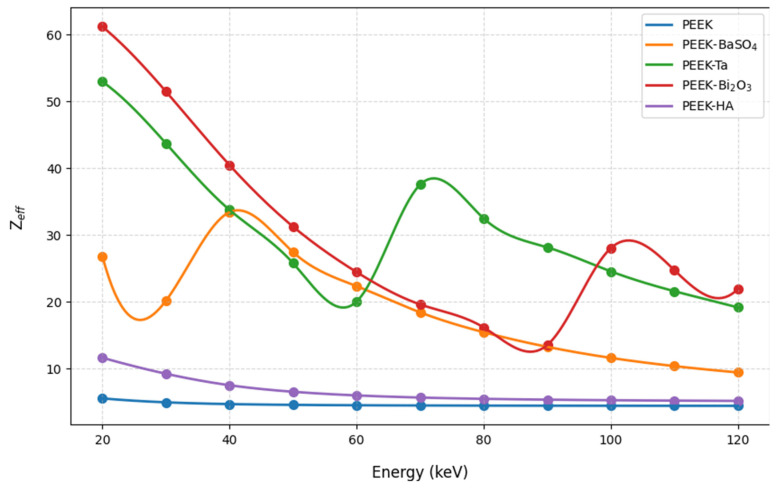
The effective atomic numbers of the studied samples within the studied energy range.

**Figure 4:**
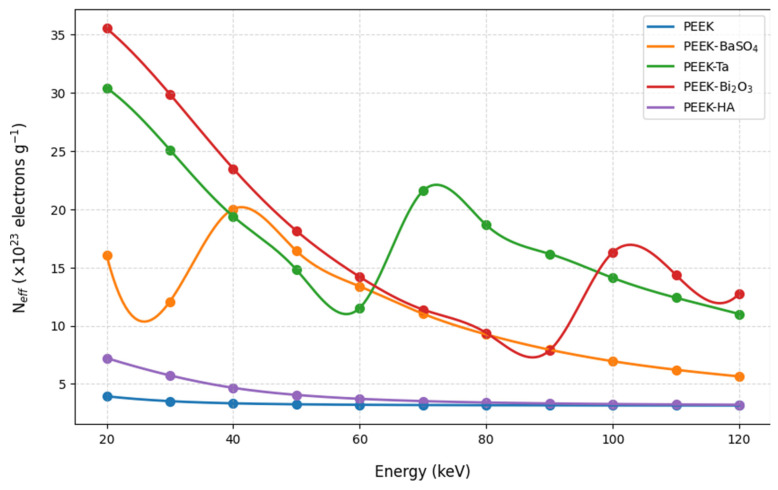
The effective electron densities of the studied samples within the studied energy range.

The EpiXS results of photon attenuation within the studied materials are shown in Figure 2, Figure 3 and Figure 4. The mass attenuation coefficients decrease with increasing photon energy for all materials, which is consistent with theoretical expectations. At lower energies, the photoelectric effect dominates, resulting in higher attenuation. The highest attenuation is observed at 20 keV, where photoelectric absorption is most prominent. As photon energy increases, Compton scattering becomes the dominant interaction, leading to a gradual decline in attenuation.

It is seen that each composite impacts the attenuation properties of PEEK differently based on the atomic number (Z) of the added material. The lowest attenuation values are observed for pure PEEK, which indicates weak X-ray attenuation.

The results show that at most studied energies, HA composites do not enhance attenuation compared to pure PEEK, which could be due to the presence of calcium.

BaSO_4_ composites led to an increase in mass attenuation coefficients ranging from approximately 9% to 14% across the diagnostic energy range (20–120 keV) compared to pure PEEK. These values were obtained by calculating the relative difference at selected photon energies (e.g., 30, 50, 80, and 100 keV) using Equation (10):

(10) $$\%Increase=\left(\frac{\mu_{PEEK+COMPOSITES}-\mu_{PEEK}}{\mu_{PEEK}}\right)$$

where *µ* represents the attenuation coefficient derived from EpiXS. The observed enhancement is more pronounced at mid-range energies where the contribution of both photoelectric and Compton interactions is significant.

The results reveal that Ta enhances the photon attenuation of PEEK between 7% and 67%, as its high atomic number enhances both photoelectric and Compton interactions, making it suitable for applications requiring strong radiation shielding. Ba_2_O_3_ shows the largest gains in photon attenuation coefficients, increasing them between 14% and 100% of those of pure PEEK; its superior shielding effectiveness is due to the high photoelectric cross-section of Bi, making it highly effective for X-ray shielding applications.

In summary, PEEK-Bi_2_O_3_ exhibits the highest attenuation due to the high atomic number of bismuth, making it ideal for applications requiring maximum shielding and radiopacity. PEEK-Ta provides comparable attenuation but with lower secondary radiation production, making it a balanced choice for shielding without excessive scatter. PEEK-BaSO_4_ enhances attenuation while maintaining moderate density, making it suitable for medical implants requiring a compromise between radiopacity and mechanical performance. PEEK-HA has the minimum effect on the photon attenuation of PEEK among the studied composites.

The peaks in the attenuation curves for the BaSO_4_ (the orange line) and Bi_2_O_3_ (the green line) composites appear at energies corresponding to the K-absorption edges of the heavy elements (barium and bismuth).

Figure 3 and Figure 4 show the effective atomic numbers and effective electron densities of the studied samples.

The results show that composites with high atomic numbers, namely Ta and Bi_2_O_3_, cause a large increase in both effective atomic number and effective electron density, especially at lower photon energies, but they remain significantly above neat PEEK at higher energies as well. BaSO_4_ also increases Z_eff_ and N_eff_ but not as dramatically as Ta and Bi_2_O_3_. HA shows a minimum change in both Z_eff_ and N_eff_.

These results show that tantalum and bismuth oxide elevate the effective atomic number and effective electron density of PEEK, which are important factors that help enhance X-ray attenuation and contrast in X-ray imaging. The peaks at certain energies correspond to the absorption edges of heavy elements such as Ba, Bi, and Ta.

### 3.2. X-Ray Imaging Quality

The 1,000,000 events with energies ranging from 20 to 120 keV were run on the Geant4 code, which results in the energy deposit within the different materials and other statistical results, as shown in Table 2.

The results showed that pure PEEK exhibits the lowest energy deposition of 16.144 eV, while PEEK-Bi_2_O_3_ recorded the highest Edep of 222.98 eV, followed by PEEK-BaSO_4_ with Edep of 157.28 eV and PEEK_Ta with Edep of 91.665 eV. This indicates that heavy composites (especially Bi_2_O_3_ and BaSO_4_) increase the probability of photon interactions, leading to larger energy absorption.

The root mean square (RMS) values, which represent the spread in energy deposition, are lowest in pure PEEK at 681.3 eV, with PEEK-Bi_2_O_3_ of 3.76 keV and with PEEK-BaSO_4_ of 3.041 keV showing the most pronounced spreads. A larger RMS in these composites suggests a wider distribution of energy deposition events, which is consistent with the increased interaction probability of high atomic number materials.

Dividing the RMS by the mean energy deposit will represent the fluctuation of the energy deposit; multiplying this by the square root of initial energy indicates how materials respond at different initial energies.

The results show that the energy resolution of bone is moderate, which means that natural bone absorbs energy in a stable but not optimal way compared to metal-doped PEEK composites. Pure PEEK has poor energy resolution, which means it introduces noise and variation, and PEEK-Ta is slightly better, but it is clear that adding heavy elements, such as BaSO_4_ and Bi_2_O_3_, improves energy resolution, making them better shielding materials.

The total track length, which reflects the cumulative path length of secondary particles generated by photon interactions, is the shortest for pure PEEK at 6.99 nm. In contrast, composites with fillers show increased track lengths up to 117 nm for PEEK-Bi_2_O_3_ and 82.9 nm for PEEK-BaSO_4_. Longer track lengths indicate enhanced secondary particle production, which can contribute to better delineation of structures in radiographic imaging.

In summary, the studied composites, especially Bi_2_O_3_ and BaSO_4_ with PEEK, increase the energy deposition and the RMS of energy distribution, which reflects the enhancement of photon interactions. In addition, the increased total track length in composites implies more extensive secondary particle generation. These changes are likely to improve X-ray contrast and image quality, making such PEEK-based mixtures promising candidates for radiopaque medical implants.

**Figure 5:**
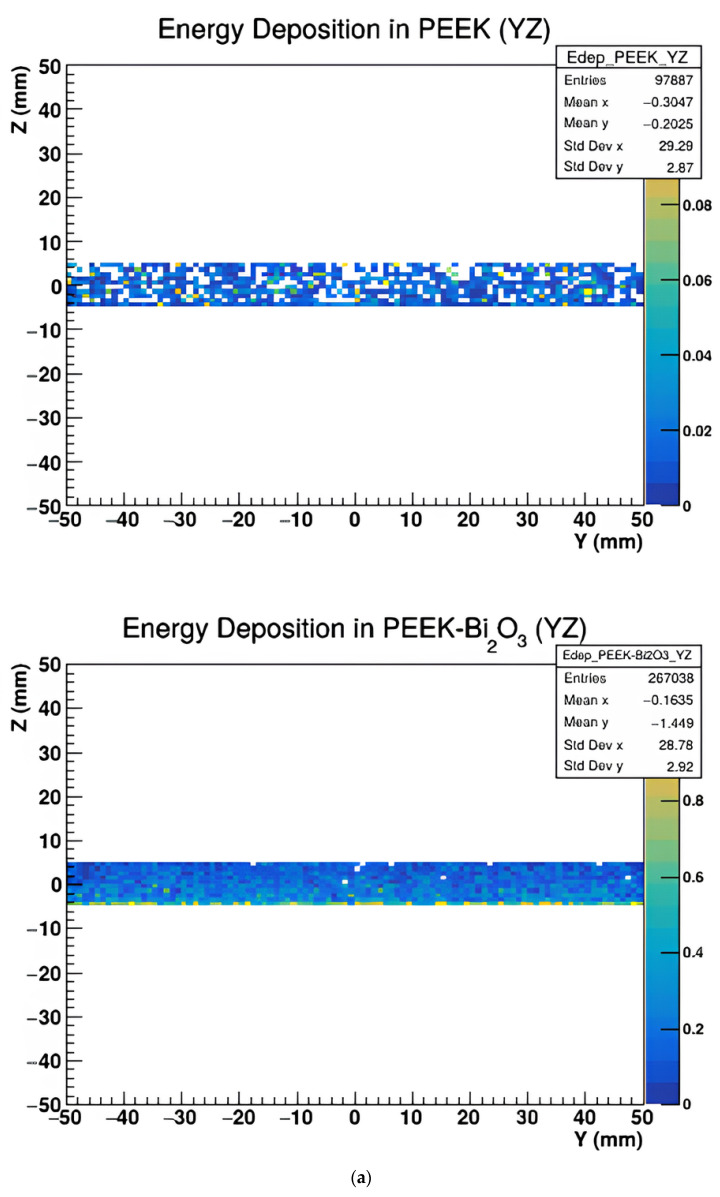

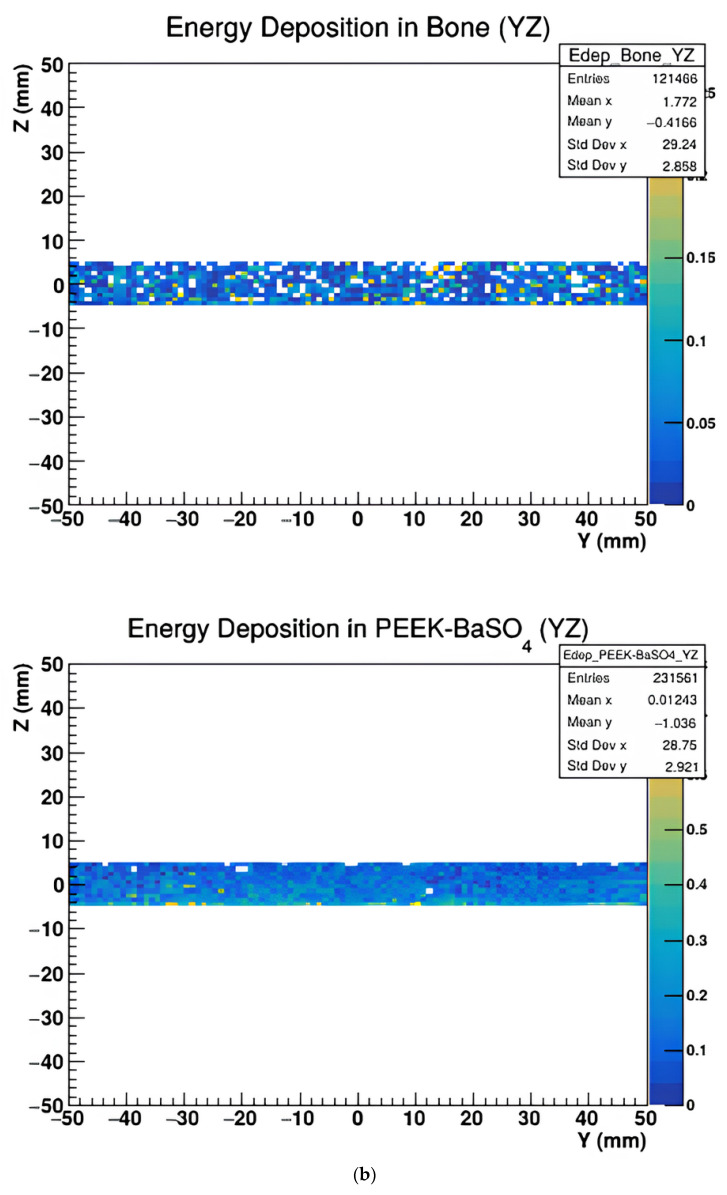

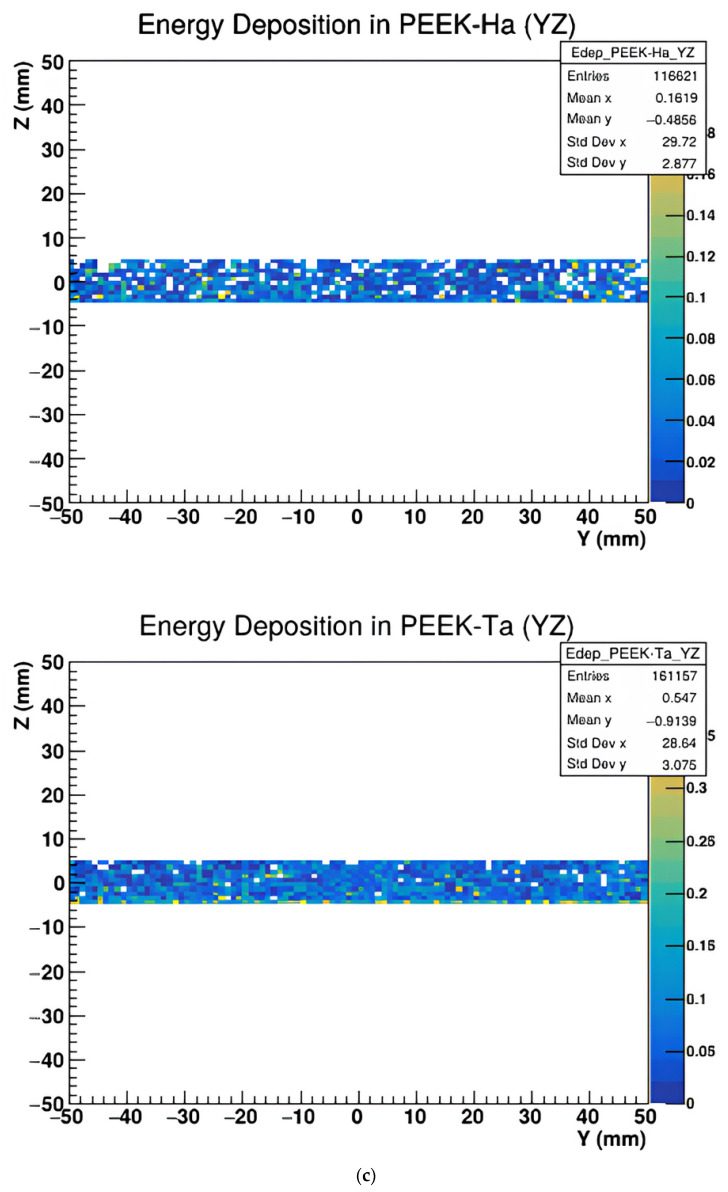
(**a**) The energy deposit map of PEEK and PEEK with Bi_2_O_3_ in the YZ plane. (**b**) The energy deposit map of Bone and PEEK with BaSO_4_ in the YZ plane. (**c**) The energy deposit map of PEEK with Ha and PEEK with Ta in the YZ plane.

**Figure 6:**
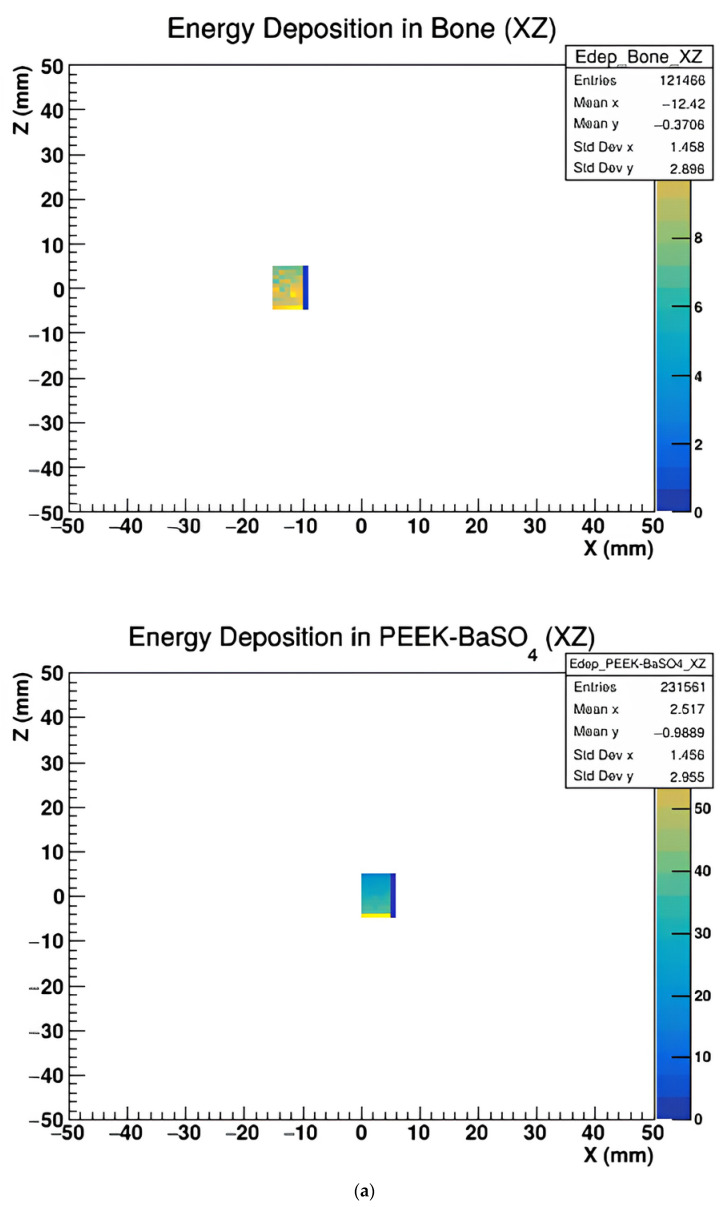

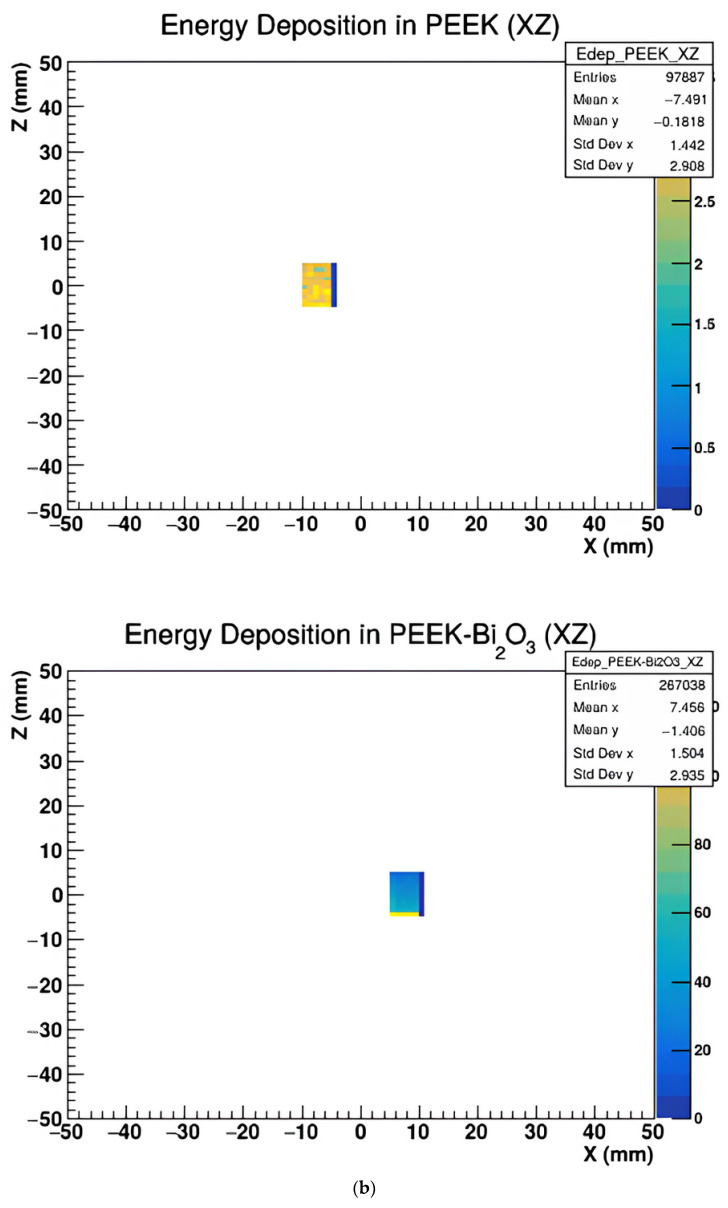

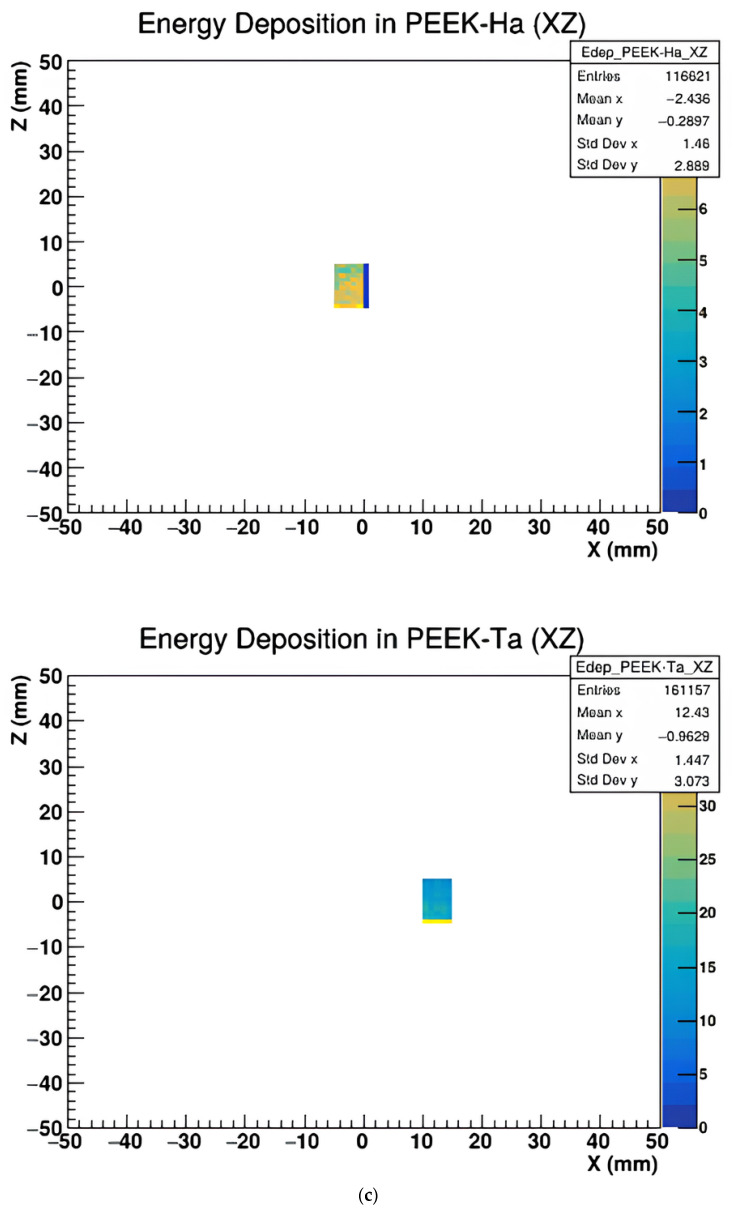
(**a**). The energy deposit map of Bone and PEEK with BaSO_4_ materials in the XZ plane. (**b**) The energy deposit map of PEEK and PEEK with Bi_2_O_3_ in the XZ plane. (**c**) The energy deposit map of PEEK with Ha and PEEK with Ta in the XZ plane.

**Figure 7:**
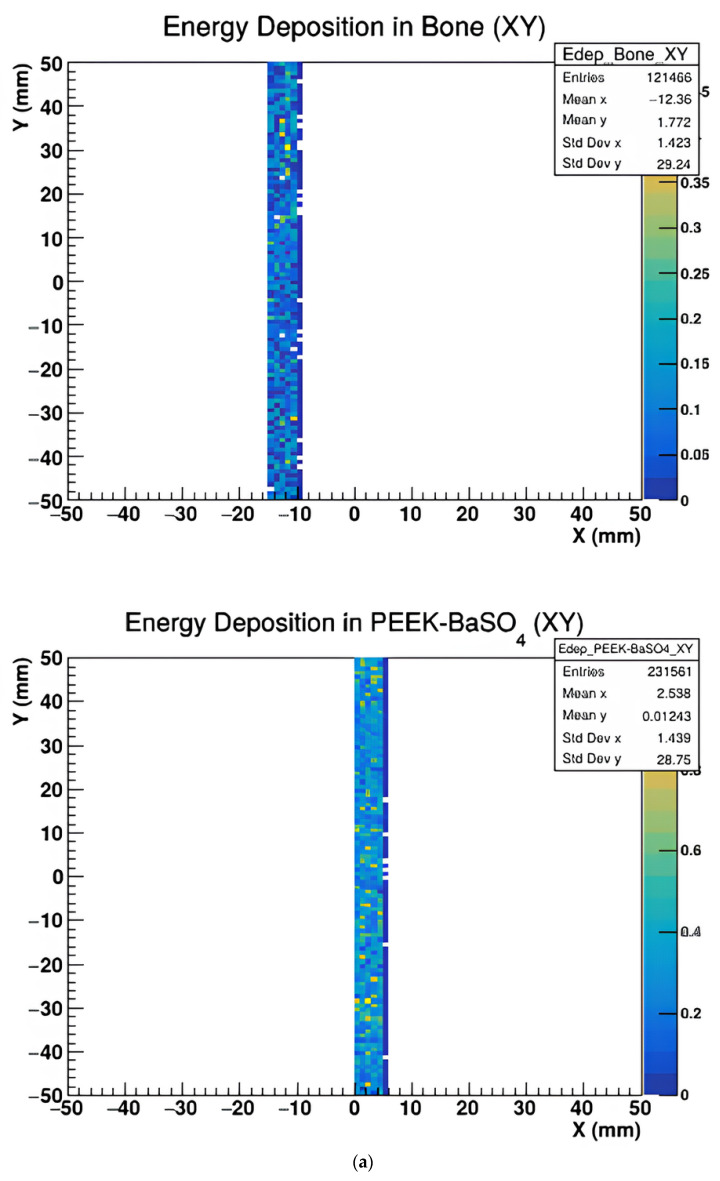

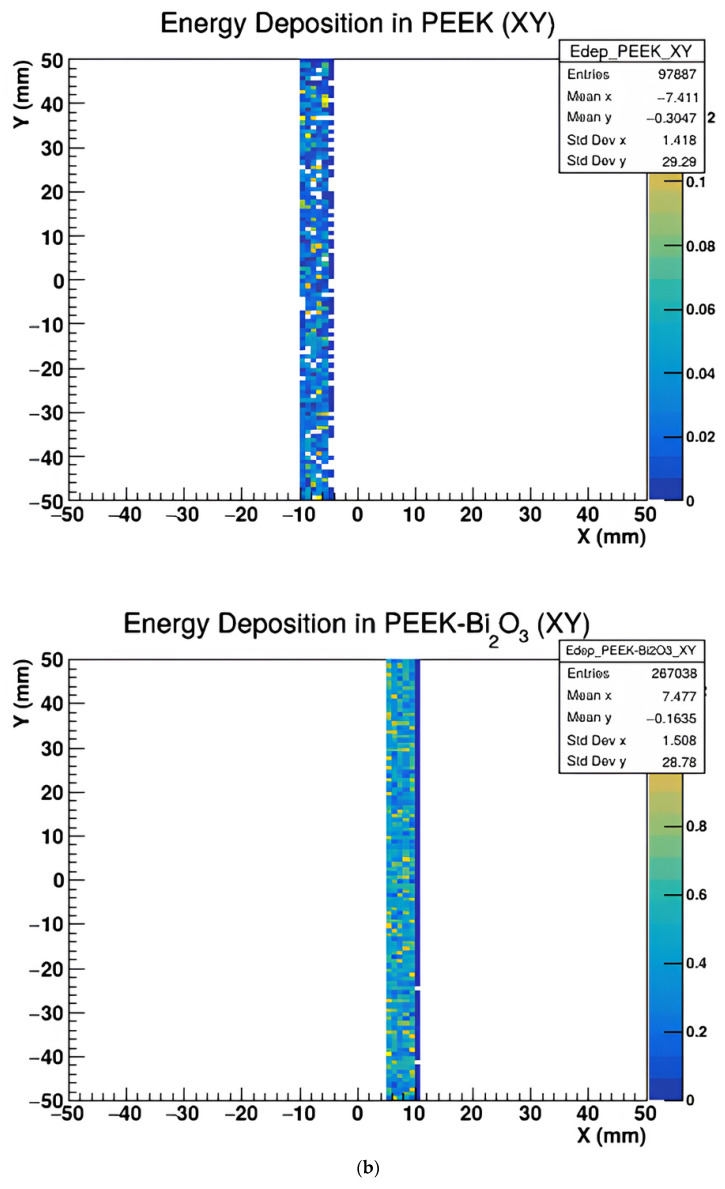

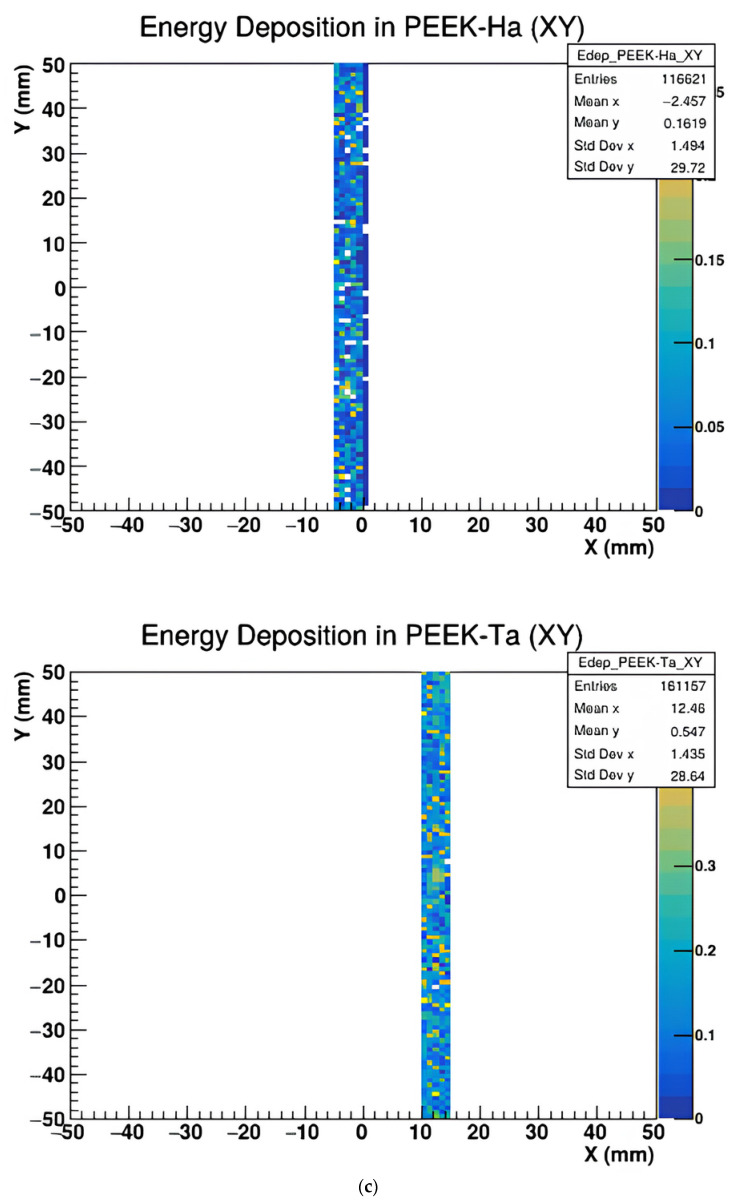
(**a**) The energy deposit map of Bone and PEEK with BaSO_4_ materials in the XY plane. (**b**) The energy deposit map of PEEK and PEEK with Bi_2_O_3_ in the XY plane. (**c**) The energy deposit map of PEEK with Ha and PEEK with Ta in the XY plane.

Figure 5, Figure 6 and Figure 7 represent the energy deposition in different projections in all investigated samples, showing energy maps in XY, XZ, and YZ planes to assess the X-ray imaging quality of the studied samples.

The above figures show that the image quality varies with projection orientation and material type.

The SNR was found for all investigated materials, as shown in Table 3. Bone (8.73 in XY, 8.59 in XZ) and PEEK-Ta (8.68 in XY, 8.61 in XZ) show the highest SNR values, indicating these materials provide the most stable energy deposition with the least noise. PEEK-Ta has nearly identical SNR values to Bone, suggesting that it is an excellent substitute for Bone in applications where mechanical strength and radiation resistance are required. On the other hand, PEEK-HA (1.71 in XY, 1.68 in XZ) and PEEK-BaSO_4_ (1.73 in XY, 1.72 in XZ) have the lowest SNR among the tested materials, which means that they introduce variations in energy deposition. PEEK (5.23 in XY, 5.18 in XZ) and PEEK-Bi_2_O_3_ (4.99 in XY, 4.94 in XZ) show moderate SNR, which means they are better than PEEK-HA and PEEK-BaSO_4_. In conclusion, PEEK and PEEK-Bi_2_O_3_ provide moderate energy stability, providing a balance between performance and material composition while PEEK-Ta has the best energy deposition consistency, making it suitable for medical implants.

## 4. Discussion

This theoretical investigation provides a comparative evaluation of pure PEEK and PEEK with its composites, namely, Bi_2_O_3_, Ta, BaSO_4_, and HA, in terms of photon interaction characteristics and X-ray imaging performance, using the EpiXS 2.0.1 software and the Geant4 simulation.

The mass attenuation coefficients represented in Figure 2 show a clear dependence on the atomic number of the filler. PEEK-Bi_2_O_3_ demonstrates the highest attenuation across the studied energy range due to the high atomic number of bismuth (Z = 83), which significantly enhances the photoelectric absorption, particularly at lower photon energies where this interaction dominates. Similarly, PEEK-Ta also exhibits enhanced attenuation, somewhat lower than Bi_2_O_3_, due to its moderate atomic number (Z = 73) and its ability to effectively balance between photoelectric absorption and Compton scattering across a broader energy range. The structured patterns observed in the attenuation curves of PEEK-BaSO_4_ and PEEK-Ta are attributed to the K-edge absorption of barium (37.4 keV) and tantalum (67.4 keV), respectively, where sudden increases in attenuation occur due to the increased probability of K-shell photoelectric interactions.

Although the study focused on the energy range from 20 to 120 keV, it is expected that attenuation coefficients would increase further at photon energies below 20 keV, particularly due to the Z^4^–Z^5^ dependence of the photoelectric effect. However, such low-energy photons are typically avoided in clinical settings due to increased patient dose and limited tissue penetration.

Figure 3 and Figure 4 further support the impact of high-Z fillers, showing substantial increases in both effective atomic number and effective electron density of PEEK-Bi_2_O_3_ and PEEK-Ta, especially at lower energies. These parameters are critical for predicting the likelihood of photon interactions. The energy-dependent peaks observed in these curves align with the absorption edges of the respective high-Z elements, confirming their contribution to enhanced attenuation and radiopacity.

In terms of imaging performance, Table 2 shows that PEEK-Bi_2_O_3_ records the highest energy deposition (222.98 eV), followed by PEEK-BaSO_4_ (157.28 eV) and PEEK-Ta (91.67 eV), while pure PEEK shows the lowest (16.14 eV). This reflects the increased probability of photon interactions in the high-Z composites. Root mean square (RMS) values of the energy deposition also increase with atomic number, indicating broader distributions of energy absorption, consistent with increased interaction cross-sections. These results suggest that Bi_2_O_3_ and BaSO_4_ fillers enhance not only the magnitude but also the complexity of energy deposition events, improving X-ray image contrast.

The total track length, which indicates the cumulative path of secondary particles generated during photon interactions, increases significantly in composites compared to pure PEEK. For instance, PEEK-Bi_2_O_3_ shows a track length of 117 nm, whereas pure PEEK exhibits only 6.99 nm. This suggests that high-Z fillers not only increase primary photon interactions but also enhance the generation of secondary radiation, further improving structural details in radiographic imaging.

Energy deposition maps shown in Figure 5, Figure 6 and Figure 7 in the XY, XZ, and YZ projections demonstrate the influence of material composition on the spatial distribution of energy absorption. PEEK-Bi_2_O_3_ and PEEK-Ta exhibit dense, high-intensity regions corresponding to localized energy absorption, while pure PEEK and PEEK-HA show more diffuse patterns with lower contrast. These visualizations confirm the superior radiopacity and contrast enhancement potential of high-Z composites.

Table 3 presents signal-to-noise ratios (SNRs), a key metric in imaging quality. PEEK-Ta exhibits SNR values nearly identical to bone in both XY and XZ projections, confirming its potential as a radiopaque material with stable imaging performance. In contrast, PEEK-HA and PEEK-BaSO_4_ display lower SNRs, indicating increased image noise and less reliable energy deposition. PEEK-Bi_2_O_3_, while providing the highest attenuation and energy deposition, exhibits moderate SNR due to potential over-attenuation or increased scatter.

Collectively, these findings demonstrate that incorporating high-Z fillers into PEEK can significantly enhance both shielding and imaging performance. PEEK-Bi_2_O_3_ is ideal for maximum attenuation and energy absorption, while PEEK-Ta offers a balanced performance with lower secondary radiation and high imaging stability. PEEK-BaSO_4_ provides moderate enhancement, and PEEK-HA contributes minimal benefit, confirming its limited applicability for radiographic purposes.

## 5. Conclusions

In conclusion, PEEK-Bi_2_O_3_ offers the best photon attenuation and the highest energy deposition, making it ideal for X-ray shielding and radiopaque medical implants, while PEEK-Ta provides a strong balance between attenuation, energy deposition consistency, and reduced secondary radiation, making it a preferred choice for applications requiring both shielding and stable imaging. PEEK-BaSO_4_ improves attenuation while maintaining moderate density, making it suitable for implants that need a balance between radiopacity and mechanical properties.

On the other hand, PEEK-HA shows minimal enhancement in photon attenuation, effective atomic number, and energy deposition, indicating limited benefit for radiographic applications.

This study provides a theoretical evaluation of PEEK with and without different composites for medical implant applications in terms of photon shielding and X-ray imaging quality. Future research can focus on experimental validations of the simulation results and the study of different weight fractions, as well as maybe mixing different composites with different weight fractions with PEEK to evaluate their effects on shielding capabilities and imaging qualities as well as mechanical and biocompatibility properties.

## Figures and Tables

**Table 1:** Properties of the studied materials.

Material	Density (g/cm^3^)	Elastic Modulus (GPa)
Pure PEEK	1.32	4
PEEK + 20% BaSO_4_	1.52	5
PEEK + 20% Bi_2_O_3_	3.84	10.5
PEEK + 20% HA	1.68	5.2
PEEK + 20% Ta	4.65	11

**Table 2:** Statistical results of the energy deposit from the Geant4 code.

Material	*E_dep_* (eV)	*RMS* (keV)	$\sqrt{E_{0}}\frac{RMS}{E_{dep}}\times 100\%$	Total Track Length
Bone	62.725	1.683	2683 Â ± 0.8483	24.9 Â ± 925
PEEK	16.144	0.6813	4220 Â ± 1.335	6.99 Â ± 467
PEEK-HA	42.346	1.291	3049 Â ± 0.9643	19.7 Â ± 854
PEEK-BaSO_4_	157.28	3.041	1934 Â ± 0.6115	82.9 Â ± 2030
PEEK-Bi_2_O_3_	222.98	3.76	1686 Â ± 0.5332	117 Â ± 2570
PEEK-Ta	91.665	2.173	2370 Â ± 0.7496	42.5 Â ± 1390

**Table 3:** The SNR from energy deposits at investigated samples of all three projections.

Projection	XY			XZ			YZ		
	Mean	Std Dev	SNR	Mean	Std Dev	SNR	Mean	Std Dev	SNR
Bone	12.3913	1.42016	8.72529	12.4317	1.44665	8.5934	1.7624	29.0168	0.060737
PEEK	7.48475	1.43181	5.22746	7.49844	1.4486	5.17635	0.137307	29.1031	0.004718
PEEK-Ha	2.5441	1.48957	1.70795	2.46441	1.47013	1.67632	0.764383	29.5334	0.02588
PEEK-BaSO_4_	2.51675	1.45083	1.73469	2.49578	1.45425	1.7162	0.0724215	28.5309	0.002538
PEEK-Bi_2_O_3_	7.50285	1.50493	4.98552	7.44557	1.5075	4.93902	0.0190219	28.5529	0.00067
PEEK-Ta	12.4418	1.43265	8.68448	12.446	1.4449	8.61373	0.0580081	29.1352	0.001991

## Data Availability

The original contributions presented in this study are included in the article. Further inquiries can be directed to the corresponding author.

## References

[1] Yang Z., Guo W., Yang W., Song J., Hu W., Wang K. (2025). Polyetheretherketone biomaterials and their current progress, modification-based biomedical applications and future challenges. Mater. Des..

[2] Abdullah M.R., Goharian A., Abdul Kadir M.R., Wahit M.U. (2015). Biomechanical and bioactivity concepts of polyetheretherketone composites for use in orthopedic implants-a review. J. Biomed. Mater. Res. Part A.

[3] Kaya N., Sasany R., Yanıkoglu N., Tosun B. (2024). Investigation of color and physicomechanical properties of peek and pekk after storage in a different medium. Sci. Rep..

[4] Najeeb S., Khurshid Z., Zohaib S., Zafar M.S. (2016). Bioactivity and Osseointegration of PEEK Are Inferior to Those of Titanium: A Systematic Review. J. Oral Implantol..

[5] Senra M.R., Marques M.d.F.V., Monteiro S.N. (2023). Poly (Ether-Ether-Ketone) for Biomedical Applications: From Enhancing Bioactivity to Reinforced-Bioactive Composites—An Overview. Polymers.

[6] Pu F., Yu Y., Zhang Z., Wu W., Shao Z., Li C., Feng J., Xue L., Chen F. (2023). Research and Application of Medical Polyetheretherketone as Bone Repair Material. Macromol. Biosci..

[7] Chen Q., Thouas G.A. (2015). Metallic implant biomaterials. Mater. Sci. Eng. R Rep..

[8] Emonde C.K., Eggers M.-E., Wichmann M., Hurschler C., Ettinger M., Denkena B. (2024). Radiopacity Enhancements in Polymeric Implant Biomaterials: A Comprehensive Literature Review. ACS Biomater. Sci. Eng..

[9] Mottu F., Rüfenacht D.A., Doelker E. (1999). Radiopaque Polymeric Materials for Medical Applications. Investig. Radiol..

[10] Koç M.M., Aslan N., Kao A.P., Barber A.H. (2019). Evaluation of X-ray tomography contrast agents: A review of production, protocols, and biological applications. Microsc. Res. Technol..

[11] Wang Q., Yu X., Chen X., Gao J., Shi D., Shen Y., Tang J., He J., Li A., Yu L. (2022). A Facile Composite Strategy to Prepare a Biodegradable Polymer Based Radiopaque Raw Material for “Visualizable” Biomedical Implants. ACS Appl. Mater. Interfaces.

[12] Basgul C., Yu T., MacDonald D.W., Siskey R., Marcolongo M., Kurtz S.M. (2018). Structure–property relationships for 3D-printed PEEK intervertebral lumbar cages produced using fused filament fabrication. J. Mater. Res..

[13] Carraro F., Bagno A. (2023). Tantalum as Trabecular Metal for Endosseous Implantable Applications. Biomimetics.

[14] Liu H., Zhang Z., Gao C., Bai Y., Liu B., Wang W., Ma Y., Yang H., Li Y., Chan A. (2020). Enhancing effects of radiopaque agent BaSO_4_ on mechanical and biocompatibility properties of injectable calcium phosphate composite cement. Mater. Sci. Eng. C.

[15] Jagdale P., Serino G., Oza G., Audenino A.L., Bignardi C., Tagliaferro A., Alvarez-Gayosso C. (2021). Physical Characterization of Bismuth Oxide Nanoparticle Based Ceramic Composite for Future Biomedical Application. Materials.

[16] Ahmed L.O., Omer R.A. (2024). Hydroxyapatite biomaterials: A comprehensive review of their properties, structures, clinical applications, and producing techniques. Rev. Inorg. Chem..

[17] Tam DK Y., Ruan S., Gao P., Yu T. (2012). High-performance ballistic protection using polymer nanocomposites. Advances in Military Textiles and Personal Equipment.

[18] Hila F.C., Asuncion-Astronomo A., Dingle CA M., Jecong JF M., Javier-Hila AM V., Gili MB Z., Balderas C.V., Lopez GE P., Guillermo NR D., Amorsolo A.V. (2021). EpiXS: A Windows-based program for photon attenuation, dosimetry and shielding based on EPICS2017 (ENDF/B-VIII) and EPDL97 (ENDF/B-VI.8). Radiat. Phys. Chem..

[19] Singh K., Singh H., Sharma V., Nathuram R., Khanna A., Kumar R., Singh Bhatti S., Singh Sahota H. (2002). Gamma-ray attenuation coefficients in bismuth borate glasses. Nucl. Instrum. Methods Phys. Res. Sect. B Beam Interact. Mater. At..

[20] Un A., Demir F. (2013). Determination of mass attenuation coefficients, effective atomic numbers and effective electron numbers for heavy-weight and normal-weight concretes. Appl. Radiat. Isot..

[21] Kaewkhao J., Laopaiboon J., Chewpraditkul W. (2008). Determination of effective atomic numbers and effective electron densities for Cu/Zn alloy. J. Quant. Spectrosc. Radiat. Transf..

[22] Agostinelli S., Allison J., Amako K., Apostolakis J., Araujo H., Arce P., Asai M., Axen D., Banerjee S., Barrand G. (2003). Geant4—A simulation toolkit. Nucl. Instrum. Methods Phys. Res. Sect. A Accel. Spectrometers Detect. Assoc. Equip..

